# Aggressive Bladder Urothelial Carcinoma in an HIV‐Positive Male With Neurogenic Bladder Dysfunction due to Spina Bifida: An Autopsy Case

**DOI:** 10.1002/iju5.70020

**Published:** 2025-03-25

**Authors:** Masahiro Ueno, Norifumi Sawada, Fumiakira Yano, Koki Shinkai, Yuta Sato, Hiroshi Shimura, Tetsuo Kondo, Takanori Mochizuki, Satoru Kira, Takahiko Mitsui

**Affiliations:** ^1^ Department of Urology Interdisciplinary Graduate School of Medicine, University of Yamanashi Chuo City Yamanashi Japan; ^2^ Department of Pathology Interdisciplinary Graduate School of Medicine, University of Yamanashi Chuo City Yamanashi Japan

**Keywords:** aggressive bladder urothelial carcinoma, cisplatin, gemcitabine, HIV infection, spina bifida

## Abstract

**Introduction:**

Patients with neurogenic bladder secondary to spinal cord injury are at increased risk of developing bladder urothelial carcinoma due to urinary tract infections. The contribution of HIV infection is unknown in this group of patients.

**Case Presentation:**

A spina bifida male patient with macrohematuria and recurrent urinary tract infection was detected with bladder carcinoma covered with soft tissue thickening and was clinically diagnosed cT3N2M1. He was also diagnosed with HIV infection. Despite the treatment with Gemcitabine and Cisplatin, he developed infective endocarditis and a cerebral infarction. He died 3 months after the initiation of chemotherapy.

**Conclusions:**

This case highlights a rare case of aggressive bladder carcinoma developed in an HIV‐positive male with spina bifida, who had never used an indwelling catheter or intermittent catheterization. HIV‐positive men tend to have a higher incidence of bladder cancer at a younger age, and neurogenic bladder might accelerate the progression in this case.

**Summary**

- HIV‐positive patients with neurogenic bladder dysfunction should be followed carefully by urologists for early detection of urothelial carcinoma.

**Abbreviations**
AIDS: Acquired immunodeficiency syndrome
CD4: cluster of differentiation 4
CT: computed tomography
HIV: human immunodeficiency virus
UTI: urinary tract infection

## 1 Introduction

As modern HIV treatment has achieved an increased life expectancy, the more non‐AIDS‐related malignancies are to be seen. Carcinoma represents almost one third of all causes of deaths among HIV‐infected patients nowadays [1]. Urothelial carcinoma is one of the most common malignancies worldwide, and thus, bladder carcinoma is one of the concerns in HIV‐infected patients [2]. Patients with neurogenic bladder secondary to spinal cord injury with long‐term indwelling catheter are known to be at risk of bladder urothelial carcinoma of the bladder and immunosuppression is a known risk factor for aggressive character. There are only two cases of bladder carcinoma in patients with spinal cord injury and HIV infection [3, 4]. We present a case of bladder carcinoma with neurogenic bladder due to spina bifida and HIV infection detected simultaneously during hospitalization.

## 2 Case Presentation

**FIGURE 1:**
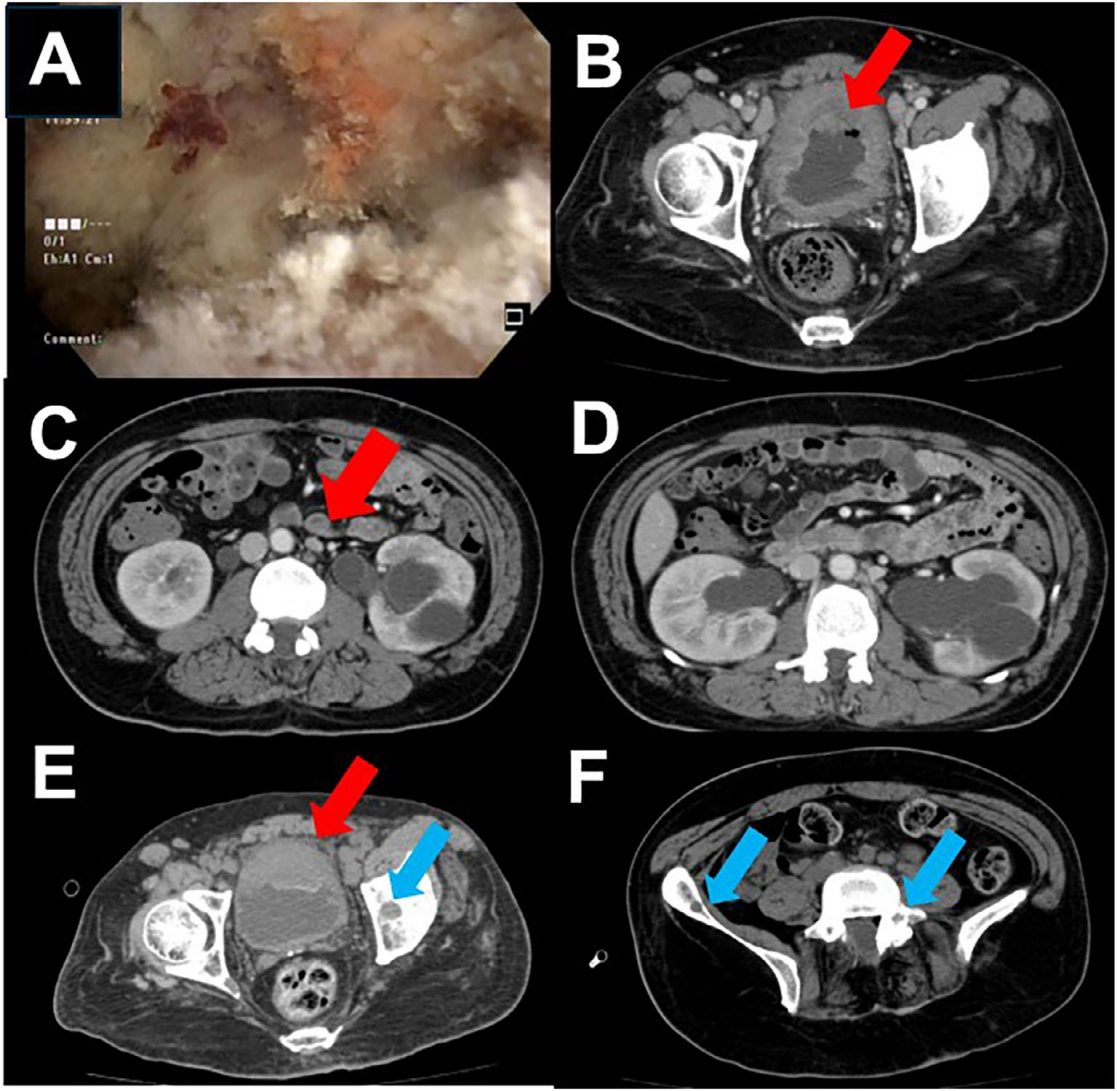
(A) Soft tissue covering the whole bladder including lesions suggesting papillary tumors. (B–D) Computed tomography revealed the cancerous lesion with infiltration of the extravesical bladder wall, multiple lymph node metastases, and bilateral hydronephroses. (E) The remaining bladder tumor (red arrow) and bone metastasis (blue arrow) are shown after two courses of gemcitabine and cisplatin. (F) Multiple pelvic bone metastases (blue arrows) appeared after chemotherapy.

A 43‐year‐old male, a company worker who is independent in his daily living activities, was referred to our hospital for macrohematuria and recurrent urinary tract infection (UTI) for 6 months prior to the referral. He had experienced recurrent UTI since the age of 10 and was diagnosed with latent spina bifida but had neither regular urological nor orthopedical check‐ups. He had used neither an indwelling catheter nor intermittent catheterization and urinated by himself. In case of UTI, he had been prescribed antibiotics in the clinic occasionally. It can be assumed that the urinary bladder suffered from chronic inflammation. To clarify the voiding function of the patient, a urodynamic test was performed, and it showed poor bladder compliance (5 mL/comH_2_O), decreased maximum bladder capacity (127 mL), and urination with abdominal pressure. To rule out malignancy in the urinary tract, cystoscopy was performed. On cystoscopy, soft tissue thickening with abnormal mucosa covering was recognized in the entire bladder with a partially papillary appearance (Figure 1A). Urine cytology revealed urothelial carcinoma, class V, suggesting high grade. Papillary tumor tissue was recognized in several lesions. The patient was a chronic heavy smoker for 20 years without occupational exposure to known carcinogenic agents. The patient was identified as HIV‐positive for the first time on the day of entering the hospital. In the first impression of the patient was a serious company financial worker living with his parents. He had been single. Careful history taking was done; he had sexual intercourse with a male partner. The patient's current presentation includes a CD4 count of 336 cells/mm^3^ and a viral load of 37 000 copies/mL.

**FIGURE 2:**
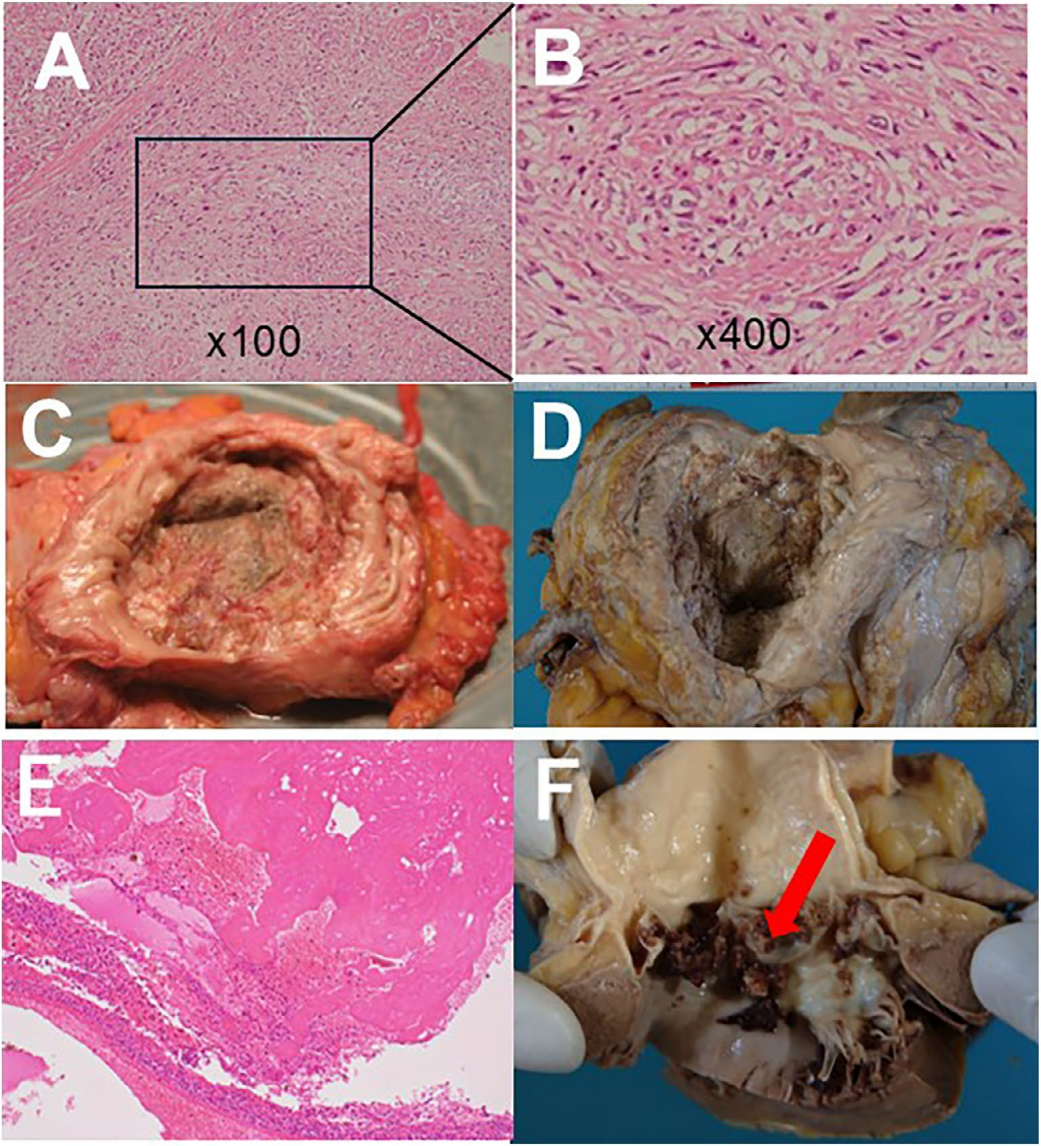
(A) Hematoxylin and eosin staining of the bladder tumor specimen showed poorly differentiated urothelial carcinoma. (B) The lesion of invasive urothelial carcinoma with sarcomatoid variant, high grade, is shown. (C) Macroscopic view of the urinary bladder specimen before formalin fixation. (D) Urinary bladder specimen after formalin fixation which shows tumor involving whole bladder. (E) Hematoxylin eosin staining of the mitral valve indicating vegetation and coagulation. (F) Vegetation in the aortic valve due to infectious endocarditis.

To validate the patient pathologically and radiographically, a transurethral resection of the bladder tumor (TURBT) was performed under general anesthesia. Pathology diagnosed the tumor as invasive urothelial carcinoma, high grade, at least pT2, with squamous metaplasia and sarcomatoid variant. Computed tomography revealed the cancerous lesion with infiltration of the extravesical bladder wall, multiple lymph node metastases, and bilateral hydronephroses (Figure 1B–D). The clinical diagnosis was bladder urothelial carcinoma of cT3N2M1. Due to the presence of extensive infiltration of the tumor and metastasis, the patient was offered chemotherapy to improve overall survival. The patient was informed that a radical cystectomy would be performed if there is an objective radiographic response. Two cycles of chemotherapy with gemcitabine (1000 mg/body) and cisplatin (70 mg/m^2^) were administered. The patient was eager for the treatment, and there was no other adverse event other than mild renal impairment and reversible myelosuppression. The tumor sizes increased in size and new multiple bone metastases have emerged after two cycles with a follow‐up CT. His general condition had deteriorated, the disease developed rapidly, and the patient's consciousness deteriorated. He had presented Trousseau syndrome, and acute cerebral infarction was diagnosed by CT. Despite the edaravone treatment, no improvement was observed and the patient deceased 3 months after the initiation of chemotherapy. With the cooperation of the patient's family, a postmortem examination was performed and revealed that bladder cancer had progressed in size, involved pelvic lymph nodes, bone marrow, and peritoneal dissemination. The patient presented cerebral and renal infarction, myocardial infarction, and pulmonary edema because of infective endocarditis (Figure 2).

## 3 Discussion

The relationship between the increased risk of urothelial carcinoma and HIV‐positive patients is unknown. It has been reported that patients with traumatic spinal cord injury develop urinary bladder carcinoma at a median age of 54.0 years [5]. Since the median age of developing bladder carcinoma was 71.1 years in male patients without spinal cord injury, spinal cord injury is related to the early development of bladder carcinoma [6].

Recent comparable study indicates that spinal cord injury patients, even without permanent catheterization, represent a considerable risk for the development of bladder carcinoma [5]. More studies are warranted to determine how often cystoscopy screening should be performed in all patients with an HIV‐positive and neurogenic bladder. Neurogenic bladder, due to its propensity to easily introduce infection, may accelerate the occurrence of bladder carcinoma. Current recommendations for the initiation of antiretroviral therapy in patients infected with HIV are based on CD4 T‐lymphocyte cell counts and plasma HIV RNA levels [7]. The patient's serum CD4 count of 336 cells/mm^3^ was less than that of a healthy male, which indicates the immunosuppressive state of the patient [8]. The viral load of 37 000 copies/mL is less than that of the average acute phase. The viral load is usually a hallmark to evaluate the effect of antiviral therapy. For untreated HIV‐infected patients, viral load is just a factor in prospecting the progress of HIV infection [9]. At our hospital, this patient was carefully followed by the infection control unit, and antiviral therapy was suggested to start considering the HIV RNA levels and the viral load. HIV‐positive men tend to have a higher incidence of bladder cancer at a younger age, but the extent to which this contributes to this case is unclear, and further investigation is needed.

Trousseau syndrome is a well‐known malignancy‐associated hypercoagulative state, which leads to venous or arterial thrombosis [10]. It has been reported that 15% of carcinoma patients suffer a thromboembolic event during their clinical course, and almost 50% exhibit evidence of venous thromboembolism on postmortem examinations [11]. In the presented case, edaravone treatment was administered; however, there could be an option of providing systemic anticoagulation therapy [12]. There is a comparable study of extended use of heparin and darteparin (low‐molecular‐weight heparin) and darteparin can be a candidate [13]. We did not administer darteparin because there was a continuous bladder hemorrhage due to bladder carcinoma.

Early detection of the bladder carcinoma could be a solution in this patient character because there are two reports of HIV‐positive patients undergoing TURBT with no recurrence. There is a report of a similar case in an HIV‐positive man with tetraplegia and neurogenic bladder who succumbed to death after one cycle of chemotherapy. Although the indwelling catheterization has been documented to be a risk factor for bladder cancer, the case lacked long‐term catheter use as in our case, which leads to the notion that the occurrence of bladder carcinoma should be checked in patients with no long‐term catheterization. It has been widely accepted that patients with neurogenic bladders should be checked for annual screening with renal and bladder ultrasound and metabolic status; however, these check‐ups are to monitor upper urinary tract deterioration and not to screen for carcinoma. Cystoscopies are performed on patients with indwelling catheters when suspected bladder stones are present. Since the immunosuppressed patient is vulnerable to infection and develops malignancy from the inflammation, an HIV‐positive patient with neurogenic bladder should be carefully monitored. More research is warranted to determine how often a routine annual screening with cystoscopy should be performed in all patients with HIV and neurogenic bladder. According to the case series report from Gaughan et al., the clinical outcomes of urothelial carcinoma in HIV patients who received cisplatin chemotherapy died within 6 months of diagnosis [14]. Contemplating the data of the presented case, bladder carcinoma with regional or metastatic disease had a poor prognosis, and careful informed consent should be achieved before starting chemotherapy.

## 4 Conclusion

Early detection of bladder carcinoma in HIV‐positive and neurogenic bladder patients is needed to provide prompt treatment and reach possible favorable outcomes. Since some of the latent spina bifida patients do not complain of urinary symptoms, urologists should enlighten primary care clinicians to ensure that patients in this cohort consult with urologists.

## Ethics Statement

This study is approved by our institutional ethical review board and the approval number is 2616.

## Consent

Informed consent was obtained from the patient's family.

## Conflicts of Interest

Norifumi Sawada and Takahiko Mitsui are the Editorial Board members of the International Journal of Urology and the co‐authors of this article. To minimize bias, they were excluded from all editorial decision‐making related to the acceptance of this article for publication.
